# Supplementary material for: Impacts of marine heatwaves on top predator distributions are variable but predictable
Source: Nat Commun. 2023 Sep 5;14:5188. doi: 10.1038/s41467-023-40849-y (PMC10480173; doi:10.1038/s41467-023-40849-y)
Supplement: Supplementary file 1 — Supplementary Information [file 41467_2023_40849_MOESM1_ESM.pdf]

Supplementary Materials for

Impacts of marine heatwaves on top predator distributions are variable but predictable

Heather Welch[1,2*], Matthew S. Savoca[3], Stephanie Brodie[1,2], Michael G. Jacox[1,2,4], Barbara A. Muhling[2,5], Thomas A. Clay[1,2,6], Megan A. Cimino[1,2], Scott R. Benson[7,8], Barbara A. Block[3], Melinda G. Conners[9], Daniel P. Costa[2,10], Fredrick D. Jordan[9], Andrew W. Leising[1], Chloe S. Mikles[3], Daniel M. Palacios[11,12], Scott A. Shaffer[13], Lesley H. Thorne[9], Jordan T. Watson[14,15], Rachel R. Holser[2], Lynn Dewitt[1], Steven J. Bograd[1,2], Elliott L. Hazen[1,2,3]

[*] Corresponding author: heather.welch@noaa.gov
[1] NOAA, Southwest Fisheries Science Center, Environmental Research Division, Monterey, CA, USA
[2] Institute of Marine Science, UC Santa Cruz, Santa Cruz, CA, USA
[3] Hopkins Marine Station, Stanford University, Pacific Grove, CA, USA
[4] NOAA, Physical Sciences Laboratory, Boulder, CO, USA
[5] NOAA Southwest Fisheries Science Center, Fisheries Resources Division, San Diego, CA, USA
[6] People and Nature, Environmental Defense Fund, Monterey, CA, USA
[7] NOAA, Southwest Fisheries Science Center, Marine Mammal and Turtle Division, Moss Landing, CA, USA
[8] Moss Landing Marine Laboratories, San Jose State University, Moss Landing, CA, USA
[9] School of Marine and Atmospheric Sciences, Stony Brook University, Stony Brook, NY, USA
[10] Department of Ecology and Evolutionary Biology, UC Santa Cruz, Santa Cruz, CA, USA
[11] Marine Mammal Institute, Oregon State University, Newport, Oregon, USA
[12] Department of Fisheries, Wildlife, and Conservation Sciences, Oregon State University, Newport, Oregon, USA
[13] Department of Biological Sciences, San Jose State University, San Jose, CA USA
[14] NOAA, Alaska Fisheries Science Center, Auke Bay Laboratory, Juneau, AK, USA
[15] Pacific Islands Ocean Observing System, University of Hawai‘i Mānoa, Honolulu, HI, USA

Table of contents

Supplementary methods
1.1. Environmental datasets
1.2. Marine heatwave conditions
1.3. Telemetry datasets
1.4. Novel validation datasets
1.5. Species distribution models
1.6. Quantifying marine heatwave impacts
1.7. Cross-jurisdictional impacts

Supplementary tables
Supplementary Table 1. Metadata for variables used in species distribution models
Supplementary Table 2. Dimensions of the novel validation datasets
Supplementary Table 3. Model performance metrics
Supplementary Table 4. Summary of species impacts
Supplementary Table 5. Sensitivity of habitat metrics to data used in model fitting.
Supplementary Table 6. Linear mixed models of variance within vs among heatwaves
Supplementary Table 7. Sensitivity of core habitat redistribution across jurisdictions to data used in model fitting

Supplementary figures
Supplementary Figure 1. Time-series of temperature anomalies
Supplementary Figure 2. Maps of baseline conditions and anomalies for key oceanographic variables
Supplementary Figure 3. Anomalies of key oceanographic variables within species 75th percentile kernels
Supplementary Figure 4. Geopolitical boundaries within the study area
Supplementary Figure 5. Relative importance of variables in the boosted regression trees
Supplementary Figure 6. Ratio of observed to predicted presences
Supplementary Figure 7. Model validation on novel data
Supplementary Figure 8. Laysan albatross distribution across datasets and years
Supplementary Figure 9. Maps of species baseline and marine heatwave distributions
Supplementary Figure 10. Species case studies of heatwave impacts
Supplementary Figure 11. Sensitivity of habitat metrics to centroid definition

1. Supplementary Materials

1.1 Environmental datasets

Daily dynamic environmental variables were acquired from Copernicus Marine Environmental Monitoring Service (https://marine.copernicus.eu/) from 2000-2020 (Supplementary Table 1). Dynamic variables used to fit species distribution models (SDMs) included primary productivity, oxygen, sea surface temperature (SST) and its spatial standard deviation, sea level anomaly, eddy kinetic energy, mixed layer depth, and chlorophyll-a. All environmental variables were resampled from their native resolutions to 0.25 degrees to match the coarsest resolution of the environmental datasets (sea surface height products) in R (version 4.0.4, R Core Team 2021), and all analyses described below were also completed in R. To account for geolocation uncertainty in the animal telemetry datasets[1] (Section 1.3.), we smoothed the resampled environmental variables by 1.25 degrees (e.g., each quarter degree pixel was re-calculated as the spatial mean of all pixels within a 1.25 degree surrounding square). The standard deviation of sea surface temperature, a common proxy for temperature fronts[2], was calculated as the spatial standard deviation within a 1.25 degree surrounding square. Chlorophyll-a was log10 transformed to account for the right-skewed distribution. Primary productivity was averaged across the upper 200 m of the water column as a proxy for foraging biomass available within the euphotic zone. Oxygen concentration at 200 m approximated the location of the eastern Pacific Oxygen Minimum Zone, which can limit vertical movements for predators capable of foraging at depth[3]. Daily eddy kinetic energy was calculated from the meridional and zonal components of geostrophic velocity anomalies.

Additional environmental variables included bathymetry, rugosity, and day of year (Supplementary a Table 1). Bathymetry was acquired from ETOPO1, and rugosity was calculated as the spatial standard deviation of bathymetry within a 1.25 degree surrounding square to capture seafloor complexity. Day of year was a sequential number between one and 366 (days between January 1st and December 31st on leap years, respectively) to account for seasonal movements of migratory predators.

Supplementary Table 1. Metadata for variables used in species distribution models. Dynamic variables vary at a daily time-step, static variables do not vary across time. All variables were resampled to a quarter degree resolution. Acronyms: The Group for High Resolution Sea Surface Temperature (GHRSST), Copernicus Marine Service (CMEMS), National Oceanic and Atmospheric Administration (NOAA).

	Variable	Units	Source	Provider	Notes
Dynamic	Primary productivity	mg m^{-3}	MERCATOR FREEBIORYS2V4	CMEMS	Average primary productivity concentration in the upper 200 m
	Oxygen	mmol m^{-3}	MERCATOR FREEBIORYS2V4	CMEMS	Dissolved oxygen concentration at 200 m
	Day of year	n/a	n/a	n/a	Range 1:366
	Temperature	° celcius	GHRSST	CMEMS	Sea surface temperature
	Temperature SD	° celcius	GHRSST	CMEMS	Standard deviation of sea surface temperature
	Sea level anomaly	m	SSALTO/DUACS	CMEMS	n/a
	Eddy kinetic engery	log m^2s^{-2}	SSALTO/DUACS	CMEMS	n/a
	Mixed layer depth	m	MERCATOR GLORYS12V1	CMEMS	n/a
	Chlorophyll-a	log mg m^{-3}	GlobColour	CMEMS	n/a
Static	Rugosity	m	ETOPO1	NOAA	Standard deviation of bathymetry
	Bathymetry	m	ETOPO1	NOAA	n/a

1.2 Marine heatwave conditions

Prominent marine heatwaves (MHWs) in the North Pacific occurred in 2014, 2015, 2019, and 2020 (Supplementary Fig. 1). Warm water conditions persisted across 2014 and 2015, however we elected to examine these years as distinct events due to their distinct warming patterns. Strongly positive temperature anomalies in the Gulf of Alaska appeared in October 2013, expanding to reach the US west coast in September 2014, penetrating to over 100m in depth across large portions of the region[4] (Fig. 1A). In fall of 2015, this pre-existing heatwave was amplified by a strong El Niño event, extending the spatial extent of the MHW westward towards Hawaii, increasing the depth penetration of warm anomalies, and producing the warmest tropical SST anomalies on record[5,6]. This MHW dissipated in late 2015. The 2019 and 2020 MHWs originated in the Gulf of Alaska during late spring (April-May) and reached the US west coast in September, though there were differences in their seasonality and drivers[7]. The 2020 event was the second largest MHW on record (following the 2014 event), and at its maximum extent, the 2019 event was only 6% smaller than the 2014 event[8]. Despite their comparable sizes, the depth penetration of warm anomalies was much shallower in 2019 and 2020, reaching maximum depths of 40-50m[4]. Both of these events rapidly retracted from the US west coast during October and November of their respective years, and dissipated below thresholds for marine heatwave classification[9] during December and January. Maximum aerial extents for each event were reached in September 2014, July 2015, September 2019, and September 2020.

For each year, marine heatwaves were assessed from August - October: the months in which the highest temperature anomalies were observed across the Pacific. In addition to SST (Fig. 1A), anomalies of oxygen at 200m, mean primary productivity within the upper 200 m, and surface chlorophyll-a conditions were also assessed during MHWs (Supplementary Fig. 2). These four variables were quantified within each species' 75th percentile kernel (Supplementary Fig. 3, see Section 1.3.1.). Anomalies during each MHW were calculated relative to mean conditions across August-October 2000-2020. We were precluded from using a longer baseline by the availability of satellite observations for chlorophyll-a (a covariate in the SDMs), which came online as a science-quality product in 1998.

Supplementary Figure 1. Temperature anomalies during the past two decades in the northeast Pacific (Fig. 1A). Daily sea surface temperature anomalies derived from a long-term baseline (2000 - 2020), smoothed by a rolling 30 day average. Red vertical bars mark the four MHW events analyzed in this study (August-October of 2014, 2015, 2019, and 2020). Source data are provided as a Source Data file.

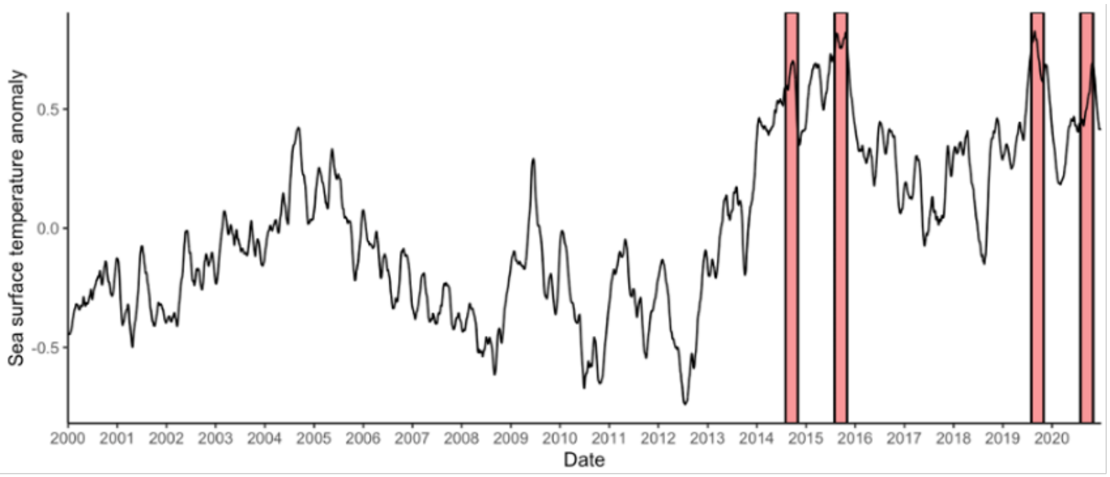

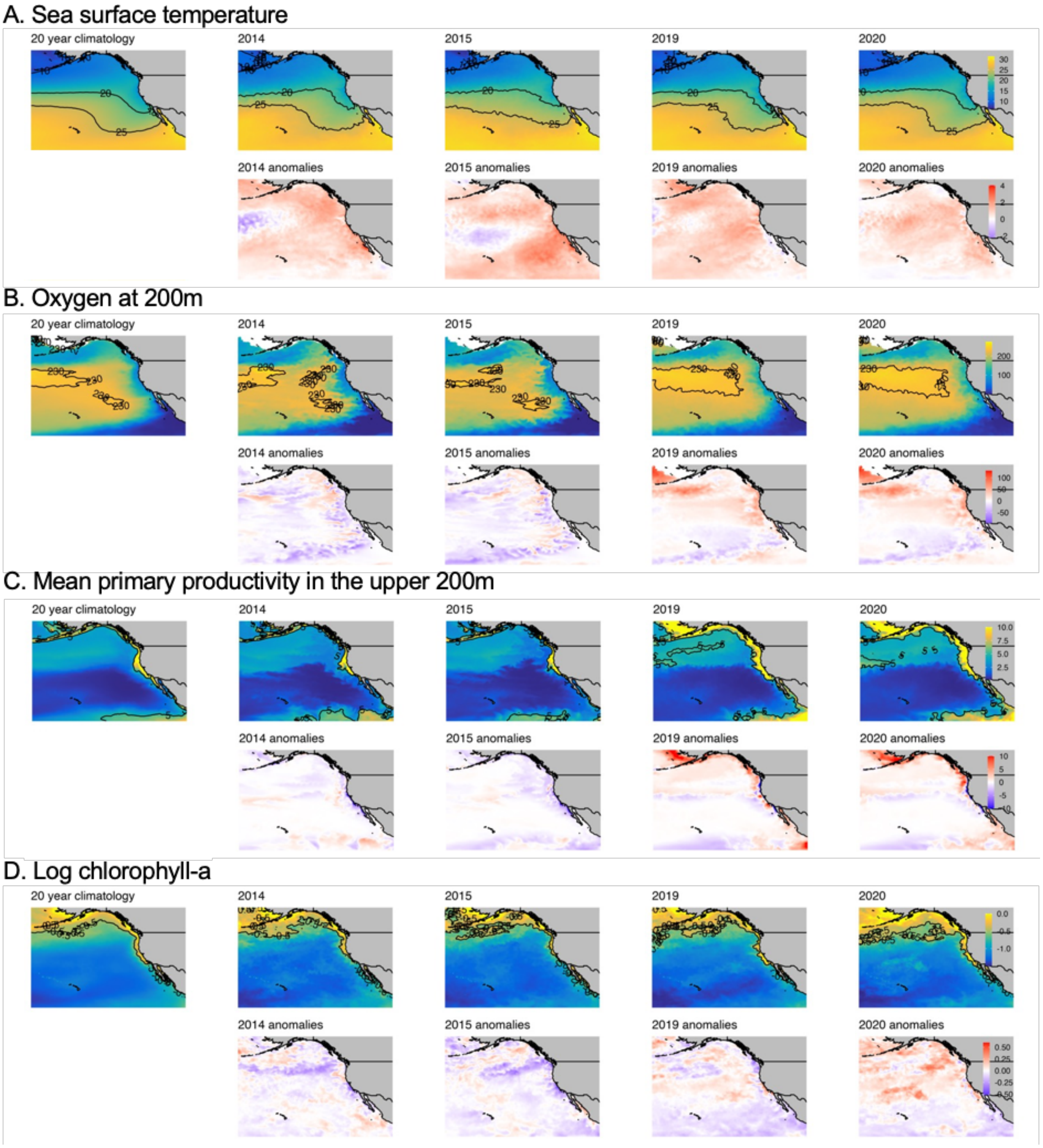

Supplementary Figure 2. Anomalies of sea surface temperature (°C; A), oxygen concentration at 200m (mmol m^{-3}; B), mean primary productivity in the upper 200m (mg m^{-3}; C), and log chlorophyll-a (mg m^{-3}; D) during each marine heatwave event. Anomalies were calculated from August-October in each year relative to a 2000-2020 August-October baseline.

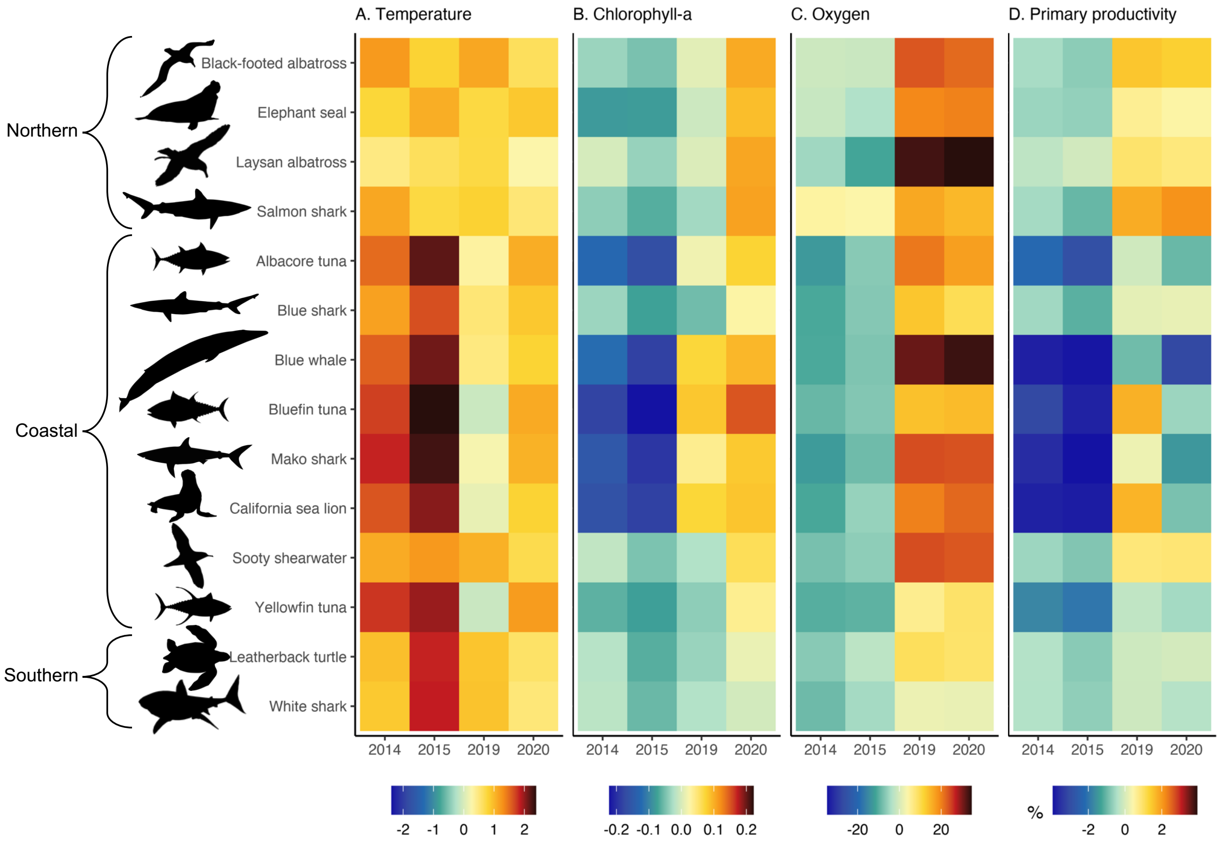

Supplementary Figure 3. Anomalies of key dynamic variables during the four marine heatwave events. Anomalies of Sea surface temperature (°C; A), log chlorophyll-a (mg m^{-3}; B), oxygen at 200m (mmol m^{-3}; C), and mean primary productivity in the upper 200m (mg m^{-3}; D) measured over the 75th percentile kernels of each species. Anomalies were calculated from August-October in each year relative to a 2000-2020 August-October baseline. Source data are provided as a Source Data file.

1.3. Telemetry datasets

Telemetry data used to build the SDMs were acquired for 14 top predators tagged within t from 2000-2010, including data from the Tagging of Pacific Predators Project[1] (TOPP) and private datasets. For all species except albatrosses, detailed methods on the number of individuals tagged, handling of tagging bias, and state space modeling are included in Block et al. (2011)[1] and Winship et al. (2013)[10]. For both albatross species, TOPP data were supplemented with additional modeled light-level geolocation data[11] from the post-breeding phase to extend the temporal coverage for each species (from two and three years to seven and eight years for black-footed (*Phoebastria nigripes)* and Laysan albatross (*P. immutabilis)*, respectively[12].

Details on the selection of the 14 species suitable for modeling are included in Hazen et al. (2013)[2]. We did not consider loggerhead sea turtles (*Caretta caretta*) in this study because the data were limited to a small region in the Southern California Bight. All telemetry datasets were clipped to a bounding box of 180°W-100°E, and 10-S-62°N to focus the analysis on the northeast Pacific Ocean, as this is where the majority of the telemetry data were located and where the four MHW anomalies were greatest (Fig. 1, Supplementary Fig. 4).

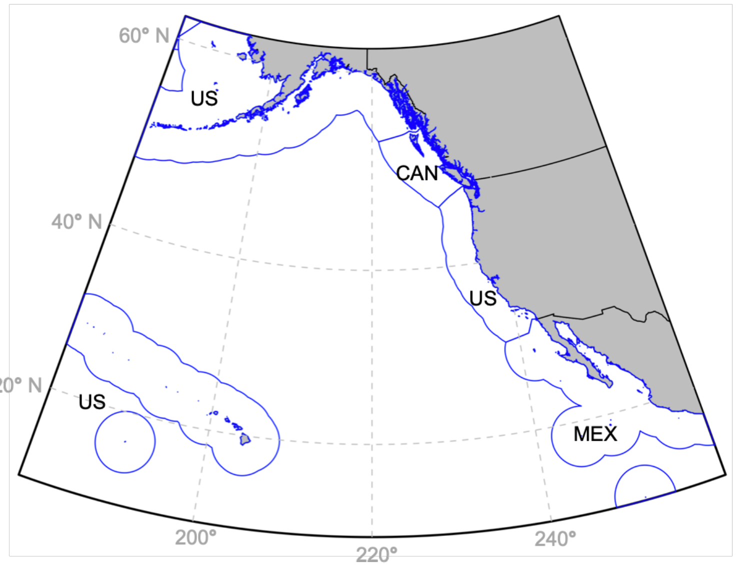

Supplementary Figure 4. Geopolitical boundaries within the study area. Lines of latitude and longitude are shown in dashed grey, Exclusive Economic Zones are shown in solid blue. Country codes: United States (US), Canada (CAN), Mexico (MEX).

The full set of available telemetry data was used for each species, as opposed to building distinct behavioral models, e.g. breeding and foraging. This decision was made for three reasons:

1) **Coarse data resolution:** the telemetry datasets from Block et al. (2011)[1] were based on geolocator data, which are regularized to one location per day and have an average error of around 200 km[10,13]. As such, the spatial scale of behavior is not sufficiently resolved to isolate meaningful foraging behaviors from tracks.

2) **Lack of separation between foraging and migrating habitat**: many of the species do not experience distinct environmental conditions during foraging and migration. For example, albatross species forage along their migration routes[14,15] as do blue whales (*Balaenoptera musculus*)[16]. Tunas target different seasonal forage resources while remaining within suitable thermal environments[17–19], adaptively following favorable environmental conditions rather than showing fidelity to particular foraging locations. As such, telemetry-based models for these top predator species frequently do not subdivide by behavior, e.g. blue sharks (*Prionace glauca*)[20], California sea lions (*Zalophus californianus*)[21], albatrosses[22].

3) **Model purpose**: this ocean-basin scale analysis aims to describe large-scale displacements in response to MHW events, rather than fine-scale behavioral partitioning. Secondly, we aim to understand how human-wildlife interactions (e.g. fisheries target catch and bycatch, ship strike) may be exacerbated during MHW events; these interactions can occur when animals are both foraging and when they are not (e.g. when in directed movement) and so we consider locations associated with all behaviors to be important when looking at range-shifts associated with MHWs.

1.3.1. Kernel densities

Species were assigned to one of three geographical groups - Northern, Coastal, and Southern - based on the locations of telemetry data collected from August-October across all years. Coastal species had over 60% of observations located within the California Current and Gulf of California Large Marine Ecosystems (blue and mako [*Isurus oxyrinchus*] sharks; albacore [*Thunnus alalunga*], yellowfin [*T. albacares*], and bluefin [*T. thynnus*] tunas; blue whales, sooty shearwaters [*Ardenna grisea*], and California sea lions), despite some of these species undertaking long migrations across the North Pacific. Northern species had observations to the northwest of Coastal species (Laysan and black-footed albatross;

elephant seals [*Mirounga angustirostris*] and salmon sharks [*Lamna ditropis*]); and Southern species had observations to the southwest (white sharks [*Carcharodon carcharias*] and leatherback turtles [*Dermochelys coriacea*]). These regional groupings were maintained throughout the analysis as MHW impacts were most similar across species within the same geographical group. Kernel densities for each geographical group were calculated by randomly subsampling each species' locational data to the same number of records, and then calculating kernel densities and the 75th percentile kernels across all species within each grouping (Fig. 1B). Kernel densities and 75th percentile kernels were also calculated for each species individually and used to quantify environmental conditions within each species' 75th percentile kernel during the MHW events (Supplementary Fig. 3).

1.4. Novel validation datasets

Independent datasets were used to validate the temporal extrapolation of telemetry-derived SDMs beyond the time-series of the training data (i.e., post-2010), particularly during MHW years (Supplementary Table 2). Datasets were acquired from a diverse range of public and government sources including fisheries observer programs, animal sightings from citizen science databases, and tagging data from dedicated programs. Data from the NOAA fisheries observer program from the California drift gillnet fishery contained records for blue, salmon, and mako shark, albacore and bluefin tuna, California sea lions, and elephant seals. Logbook data from the US troll and pole and line fisheries (1995 - 2019: see description of data in Nieto et al. 2017[23]) and NOAA archival tagging data (2003 - 2013: see description of tagging program in Childers et al. 2011[24]) contained albacore tuna records. The citizen science database eBird[25] contained records for the three seabird species, as did the North Pacific Pelagic Seabird Database[26] in addition to records for blue sharks, California sea lions, and elephant seals. Oregon State University provided records for blue whales[27], and salmon shark records were sourced from the North Pacific Groundfish Observer Program[28]. The University of California Santa Cruz elephant seal program provided records for elephant seals[29,30].

Logbook data (reported in log10 Catch Per Unit Effort (CPUE)) were filtered to exclude CPUEs of zero. For eBird data, variation in detectability was accounted for by restricting checklists to less than five hours long and five km in length, and with 10 or fewer observers according to eBird's best practices guidelines[31]. Blue whale records from Oregon State University were restricted to those with location 95% confidence levels less than 100 kilometers in radius, following Palacios et al. (2019)[27]. Species-specific convex hull polygons based on telemetry datasets were used to constrain the spatial domain of the novel validation datasets. For each species, only years with more than 30 records across all independent datasets were used for model validation. All species except for leatherback turtles, white sharks, and yellowfin tuna had at least one MHW year with adequate data for model validation (grey shading, Supplementary Table 2).

Supplementary Table 2. Dimensions of the novel validation datasets. Sources include: 1) California drift gillnet observer data, 2) US troll and pole and line logbook data, 3) National Oceanic and Atmospheric Administration tagging data, 4) opportunistic sightings from eBird, 5) survey data from the North Pacific Pelagic Seabird Database, 6) Oregon State University tagging data, 7) data from the North Pacific Groundfish Observer Program, and 8) tagging data from the University of California Santa Cruz elephant seal program. Grey shading indicates species/years with enough data for model validation (>30 records per marine heatwave (MHW) year).

		Number of records during MHW years					
**Species**	**Time-series**	**2014**	**2015**	**2019**	**2020**	**Total records**	**Sources**
Albacore tuna	2000-2019	5228	3847	5164	0	116151	1,2,3
Black-footed albatross	2000-2020	398	660	69	77	5474	4,5
Blue shark	2000-2020	41	53	11	20	1349	1,5
Blue whale	2014-2017	878	1457	0	0	5226	6
California sea lion	2000-2018	12	49	0	0	899	1,5
Elephant seal	2000-2019	120076	177440	22213	0	936222	1,5,8
Laysan albatross	2000-2020	138	129	50	23	4061	4,5
Leatherback turtle	NA	0	0	0	0	0	NA
Mako shark	2000-2020	41	13	17	34	1530	1
Bluefin tuna	2000-2020	19	37	36	12	468	1
Salmon shark	2000-2020	97	70	197	129	2329	1,7
Sooty shearwater	2000-2020	755	1165	83	530	10708	4,5
White shark	NA	0	0	0	0	0	NA
Yellowfin tuna	2000-2019	1	2	2	0	38	1

1.5. Species distribution models

1.5.1. Pseudo-absence generation

Pseudo-absences were generated using random background selection, which performs comparably or better than other selection methods, i.e. correlated random walks and buffer selection[32]. Pseudo-absences were generated at a 1:1 ratio[33] of presences for the telemetry datasets used in model fitting (section 1.3.), and novel datasets used in model validation (section 1.4.). For each presence point in both fitting and validation datasets, a pseudo-absence was generated for the same date. Species-specific convex hull polygons based on telemetry datasets were used to constrain the spatial domain of the pseudo-absences to ensure that model performance was not artificially increased by environmentally distinct pseudo-absences[32]. These convex hulls were also used to constrain the model predictions (section 1.5.5.) to ensure that models were not extrapolating beyond the spatial bounds of the training data. This spatial constraint has been previously applied to predictions based on this telemetry data, in recognition that models have unrealistic predictions when spatially-extrapolated[34].

1.5.2. Model fitting

Presence and pseudo-absence data were matched to the environmental datasets in space and time (section 1.1.). We used a boosted-regression tree (BRT) framework to model the probability of species presence as a function of the environment. BRTs are a common machine learning model, popularized by their ability to fit complex nonlinear relationships, automatic handling of collinearity effects, and their robustness to wide varieties of data types and distributions[34–37]. For all 14 species, BRTs with a binomial distribution were used to model the probability of species presence as a function of the environment. BRTs were built with a bag fraction of 0.6, and a tree complexity of three, and a learning rate that varied between 0.0001 and 0.00001 to ensure at least 2,000 trees were fit for each model[35] in addition to predicting suitable

habitat (section 1.5.5.), the relative importance of variables in each SDM was identified (Supplementary Fig. 5). Relative importance captures each variable's influence on the response[35], and reflects the number of times each variable is selected for splitting across all trees. Importance is scaled across all variables such that the sum adds to 100, producing relative importance, where higher values indicate stronger contributions. However, relative importance may not accurately reflect variable influence when variables are highly correlated.

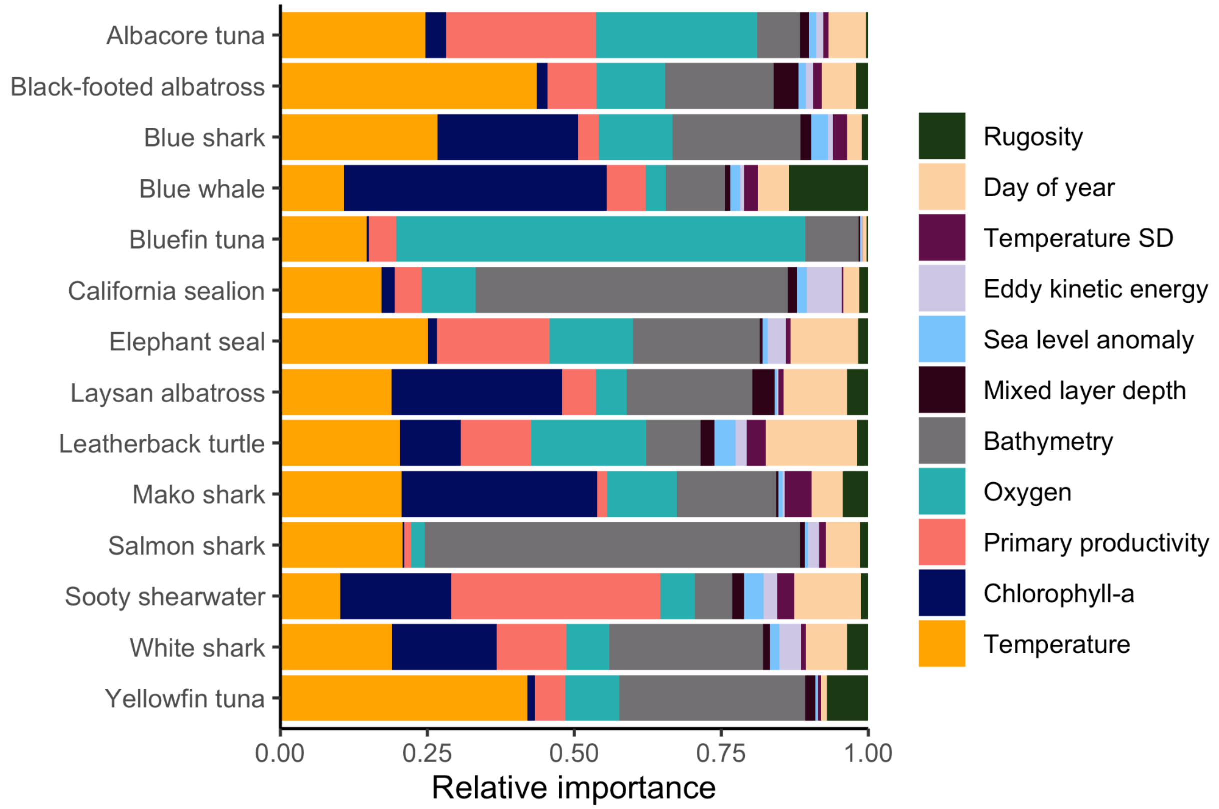

Supplementary Figure 5. Relative importance of variables in boosted regression tree models for each species. Source data are provided as a Source Data file.

1.5.3. Model performance

Three model performance metrics were evaluated: explained deviance (a measure of model explanatory power) and two measures of model predictive performance (Area Under the Receiver Operator Characteristic Curve (AUC), and True Skill Statistic (TSS)). These metrics were evaluated across the full telemetry dataset (100% validation) and using three different forms of cross-validation: 50 iterations of random 25/75% split, leave region out, and leave year out (Supplementary Table 3). For the 25/75% split, new models were trained on a random 75% selection of the telemetry data, and the remaining 25% was used to evaluate model performance. This validation was performed 50 times for each species, with each iteration selecting a new random 25/75% split. In the leave region out validation, telemetry data within three Longhurst Provinces (Eastern Pacific subarctic gyres, North Pacific polar front, North Pacific Tropical gyre; Longhurst 2007) and six Large Marine Ecosystems (Aleutian Islands, Berings Sea, Gulf of Alaska, California Current, Gulf of California, Insular Pacific-Hawaii; Sherman and Duda 1999) were each iteratively left out and used to test the performance of models built on the remaining eight regions. In the leave year out validation, telemetry data for each year was left out in iteration and used to test the performance of models built on the remaining years. These two cross-validation methods test the

sensitivity of model performance to space and time, respectively. Importantly, leave year out validation serves as a measure of how effective a prediction system of a current or developing MHW may be. For each cross-validation method, performance metrics were presented as the mean across iterations +/- one standard deviation.

Supplementary Table 3. Model performance metrics for the full dataset (A) and three different types of cross-validation (B-C). Each performance metric is presented as the mean +/- SD. Source data are provided as a Source Data file.

	**A. Full dataset**			**B. 50 iterations of 75/25 cross validation**		
	Explained deviance	TSS	AUC	Explained deviance	TSS	AUC
Albacore tuna	49.60	0.70	0.93	61.21 ± 0.40	0.77 ± 0.01	0.96 ± 0.00
Black-footed albatross	60.26	0.77	0.96	28.49 ± 0.22	0.50 ± 0.01	0.83 ± 0.00
Blue shark	34.64	0.61	0.89	49.11 ± 0.37	0.69 ± 0.01	0.92 ± 0.00
Blue whale	71.96	0.86	0.98	66.49 ± 0.41	0.81 ± 0.01	0.97 ± 0.00
Bluefin tuna	43.50	0.66	0.91	76.83 ± 0.13	0.89 ± 0.00	0.98 ± 0.00
California sea lion	37.92	0.60	0.88	72.47 ± 0.34	0.86 ± 0.01	0.98 ± 0.00
Elephant seal	33.85	0.59	0.86	33.97 ± 0.19	0.59 ± 0.01	0.86 ± 0.00
Laysan albatross	28.15	0.49	0.83	38.26 ± 0.23	0.60 ± 0.01	0.88 ± 0.00
Leatherback turtle	65.52	0.82	0.97	35.94 ± 0.41	0.61 ± 0.02	0.88 ± 0.01
Mako shark	56.81	0.75	0.95	57.13 ± 0.24	0.75 ± 0.01	0.95 ± 0.00
Salmon shark	76.74	0.89	0.98	34.64 ± 0.22	0.57 ± 0.01	0.87 ± 0.00
Sooty shearwater	48.19	0.69	0.93	51.50 ± 0.55	0.70 ± 0.02	0.93 ± 0.01
White shark	34.35	0.57	0.87	44.03 ± 0.33	0.66 ± 0.01	0.91 ± 0.00
Yellowfin tuna	65.55	0.82	0.97	65.69 ± 0.18	0.82 ± 0.00	0.97 ± 0.00

	**C. Leave space out**			**D. Leave year out**		
	Explained deviance	TSS	AUC	Explained deviance	TSS	AUC
Albacore tuna	61.54 ± 5.37	0.40 ± 0.06	0.74 ± 0.04	63.45 ± 7.35	0.76 ± 0.22	0.93 ± 0.07
Black-footed albatross	28.43 ± 5.25	0.23 ± 0.12	0.64 ± 0.09	28.63 ± 0.69	0.44 ± 0.07	0.80 ± 0.04
Blue shark	48.61 ± 3.81	0.46 ± 0.34	0.73 ± 0.18	49.73 ± 1.39	0.54 ± 0.12	0.82 ± 0.08
Blue whale	65.91 ± 3.26	0.47 ± 0.12	0.78 ± 0.09	67.39 ± 2.18	0.69 ± 0.10	0.90 ± 0.05
Bluefin tuna	69.78 ± 15.53	0.55 ± 0.23	0.77 ± 0.09	76.89 ± 0.99	0.86 ± 0.08	0.97 ± 0.04
California sea lion	84.53 ± 17.85	0.62 ± 0.24	0.85 ± 0.13	72.98 ± 1.46	0.78 ± 0.10	0.94 ± 0.04
Elephant seal	34.66 ± 2.12	0.46 ± 0.17	0.78 ± 0.07	34.14 ± 0.50	0.56 ± 0.13	0.83 ± 0.07
Laysan albatross	38.71 ± 3.57	0.40 ± 0.10	0.73 ± 0.07	38.41 ± 1.34	0.57 ± 0.08	0.86 ± 0.04
Leatherback turtle	40.91 ± 12.80	0.45 ± 0.27	0.76 ± 0.12	38.05 ± 2.96	0.37 ± 0.13	0.74 ± 0.08
Mako shark	53.69 ± 5.45	0.45 ± 0.19	0.78 ± 0.12	57.44 ± 2.35	0.74 ± 0.11	0.94 ± 0.04
Salmon shark	35.05 ± 2.59	0.44 ± 0.24	0.77 ± 0.12	34.75 ± 0.64	0.54 ± 0.05	0.85 ± 0.03
Sooty shearwater	50.65 ± 4.56	0.41 ± 0.34	0.72 ± 0.19	69.37 ± 23.07	0.18 ± 0.12	0.59 ± 0.08
White shark	48.15 ± 12.66	0.43 ± 0.35	0.68 ± 0.21	44.18 ± 0.86	0.55 ± 0.09	0.84 ± 0.06
Yellowfin tuna	62.01 ± 6.49	0.47 ± 0.25	0.73 ± 0.11	66.15 ± 0.95	0.80 ± 0.08	0.95 ± 0.02

Additionally, we calculated the daily ratio of observed to predicted values at presences from the telemetry datasets used in model fitting (Supplementary Fig. 6) to evaluate the predictive capabilities of models in a near-real time framework. Each presence was assigned a 1 and then summed to calculated observed values at presences. To calculate predicted values at presences, model predicted values at each presence were summed. Ratios of 1 indicate perfect agreement between observed and predicted presences (i.e. all model predictions at presences were 1s), while ratios above and below one indicate under and over prediction, respectively[38]. Ratios showed high alignment between daily observed and predicted presences (mean 1.5 +/- 1.3 SD), indicating strong capacity to generate predictions in a near-real time framework.

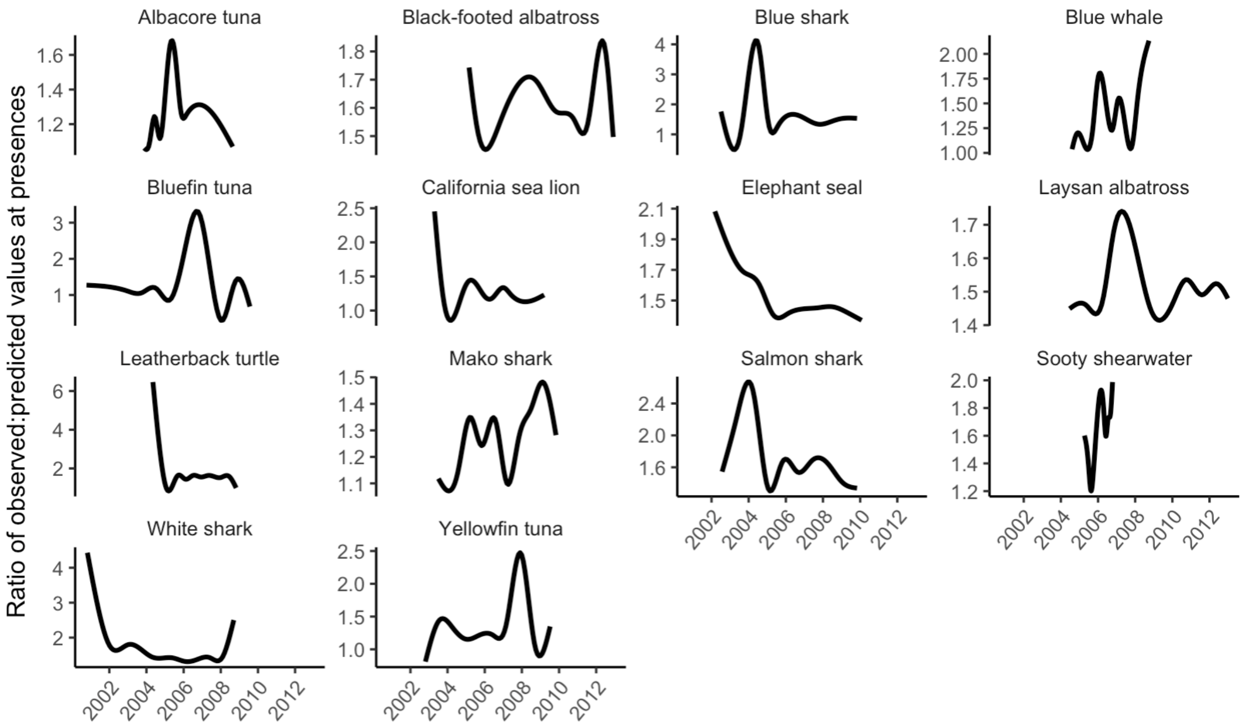

Supplementary Figure 6. The daily ratio of observed to predicted presences for each of the boosted regression tree models. Ratios of 1 indicate perfect agreement between observed and predicted presences, while ratios above and below one indicate under and over prediction, respectively. Source data are provided as a Source Data file.

1.5.4. Model validation on novel data

The telemetry data used in model fitting ended in 2010; as such, it was important to test model performance on novel data collected post-2010 (and during MHW years when possible) in order to justify temporal extrapolation. The BRT models were applied to the novel validation data and pseudo-absences, and annual AUC values were calculated to observe model performance beyond the temporal bounds of the training data (Supplementary Fig. 7). Some sampling methods closely aligned with that used to collect the telemetry data used in the models (e.g. blue whales), however others such as opportunistic sightings and shipboard surveys covered different spatial domains than the telemetry datasets and typically had lower predictive performance (Supplementary Fig. 8).

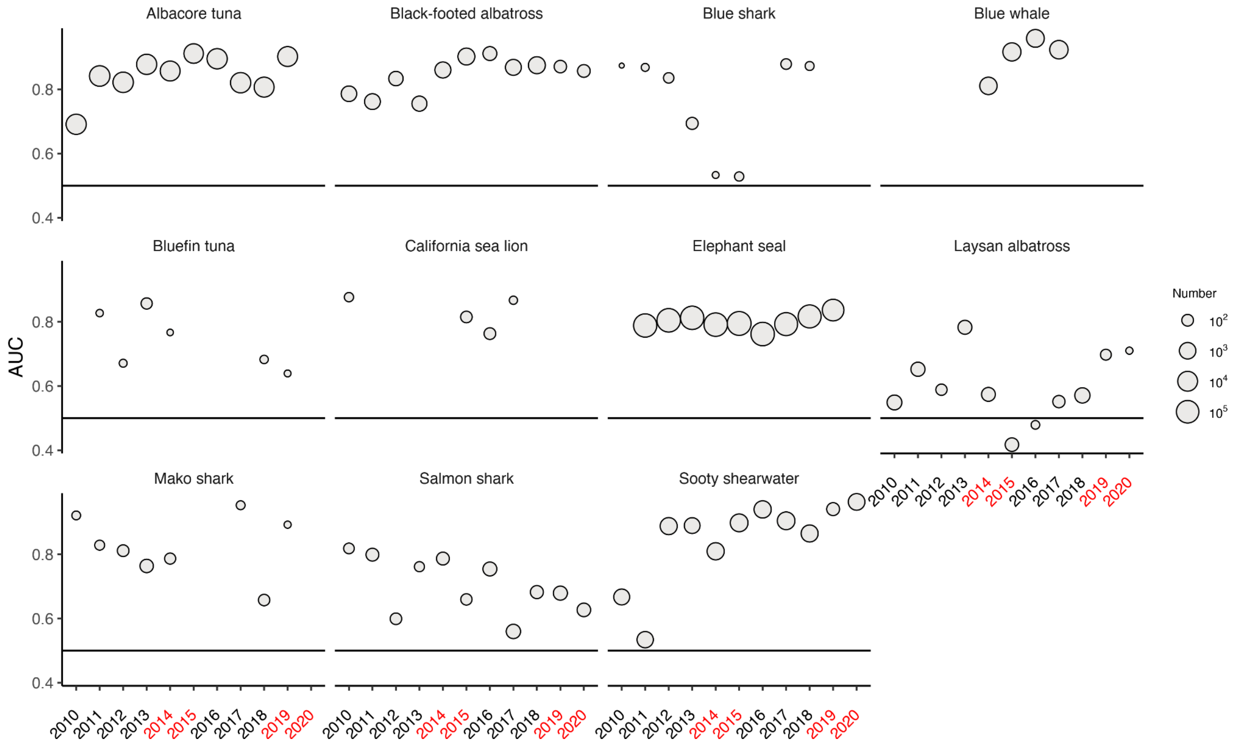

Supplementary Figure 7. Model validation on novel data from government, public, and private sources (see Methods). Plot shows annual Area Under the Receiver Operator Characteristic Curve (AUC) values for each species/year with over 30 records. Circle size indicates the number of records in each year for each species (see Supplementary Table 2 for description of datasets); horizontal line at 0.5 indicates predictive performance better and worse than random (above and below this threshold, respectively). Marine heatwave years are indicated in red. Across all species, a two-sided t-test indicated there was no significant difference in AUC between marine heatwave years (mean= 0.78) and normal years (mean=0.78); t(52.20) = -0.27, p-value = 0.791. Source data are provided as a Source Data file.

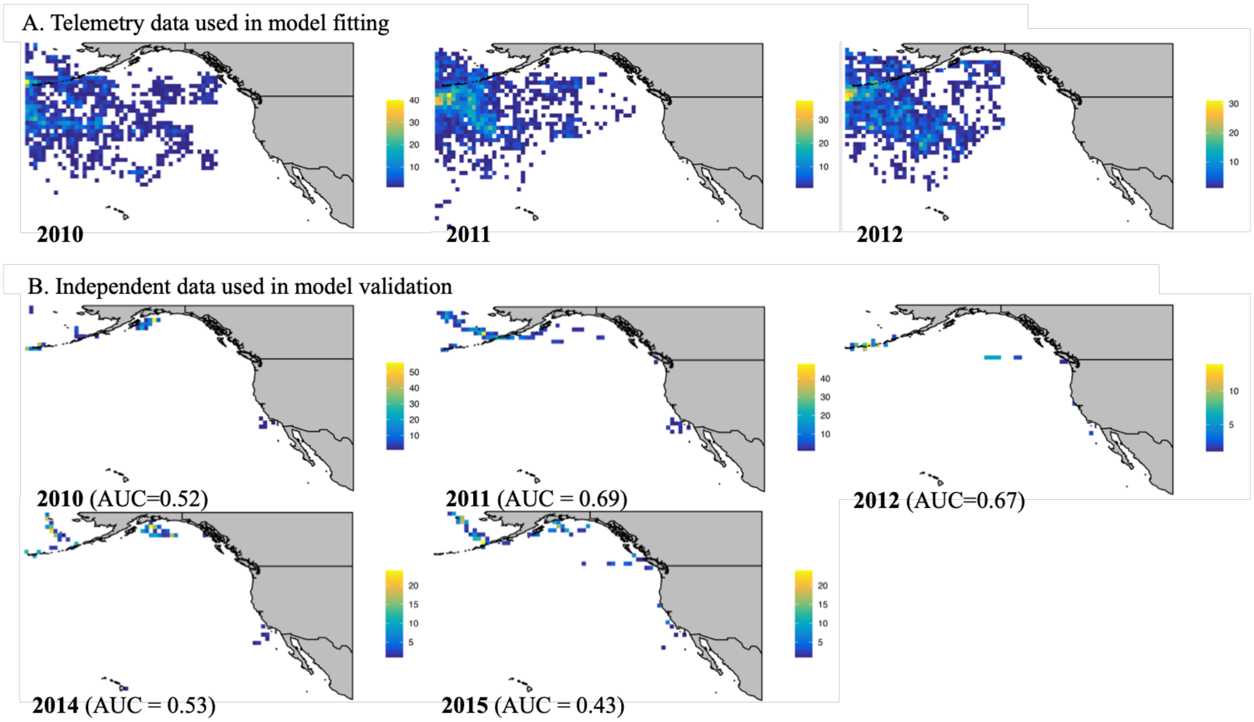

Supplementary Figure 8. Comparison of Laysan albatross distributions from A. telemetry data used in model fitting, and B. independent datasets used in model validation (opportunistic sightings from eBird and survey data from the North Pacific Pelagic Seabird Database) during overlapping years (2010-12) and two marine heatwave years (2014, 2015). Telemetry data shows relatively low utilization in the Bering Sea and Southern California Bight, resulting in lower AUC value during years with more validation data in those areas (2010, 2014, 2015). Color bar indicates the number of records.

1.5.5. Temperature-only models

In addition to multivariable boosted regression tree models, we fit SST-only models for each species using the same methodology (section 1.5.2.) to evaluate differences in performance. SST-only models were predicted over novel validation data and pseudo-absences and annual AUC values were calculated as in section 1.5.4. AUCs from the multivariable and SST-only models were evaluated for significant difference using a two-sided t-test.

1.5.6. Model prediction

The BRT models were predicted over the daily environmental data from 2000-2020 (section 1.1.), and spatially-constrained within the convex hull of the training data. The resultant habitat suitability predictions ranged from 0-1, with low and high values indicating unsuitable and suitable habitat, respectively. These continuous predictions were averaged to create an August-October 2000-2020 mean, and August-October anomalies during each MHW were calculated relative to the long-term mean (Supplementary Fig. 9). In addition to continuous habitat suitability, binary core habitat was also calculated for each day. Core habitat was defined as pixels with habitat suitability greater than or equal to the top 50% of predicted values at true presences (i.e. models were predicted back on the telemetry data used in model fitting, and 50% prediction quantile was used as the threshold). The 50% threshold was selected over the more conservative thresholds used in the climate projection literature (e.g. 25%, 30%[2,39]) as these conservative thresholds sometimes resulted in no core habitat during extreme events (e.g. 2015).

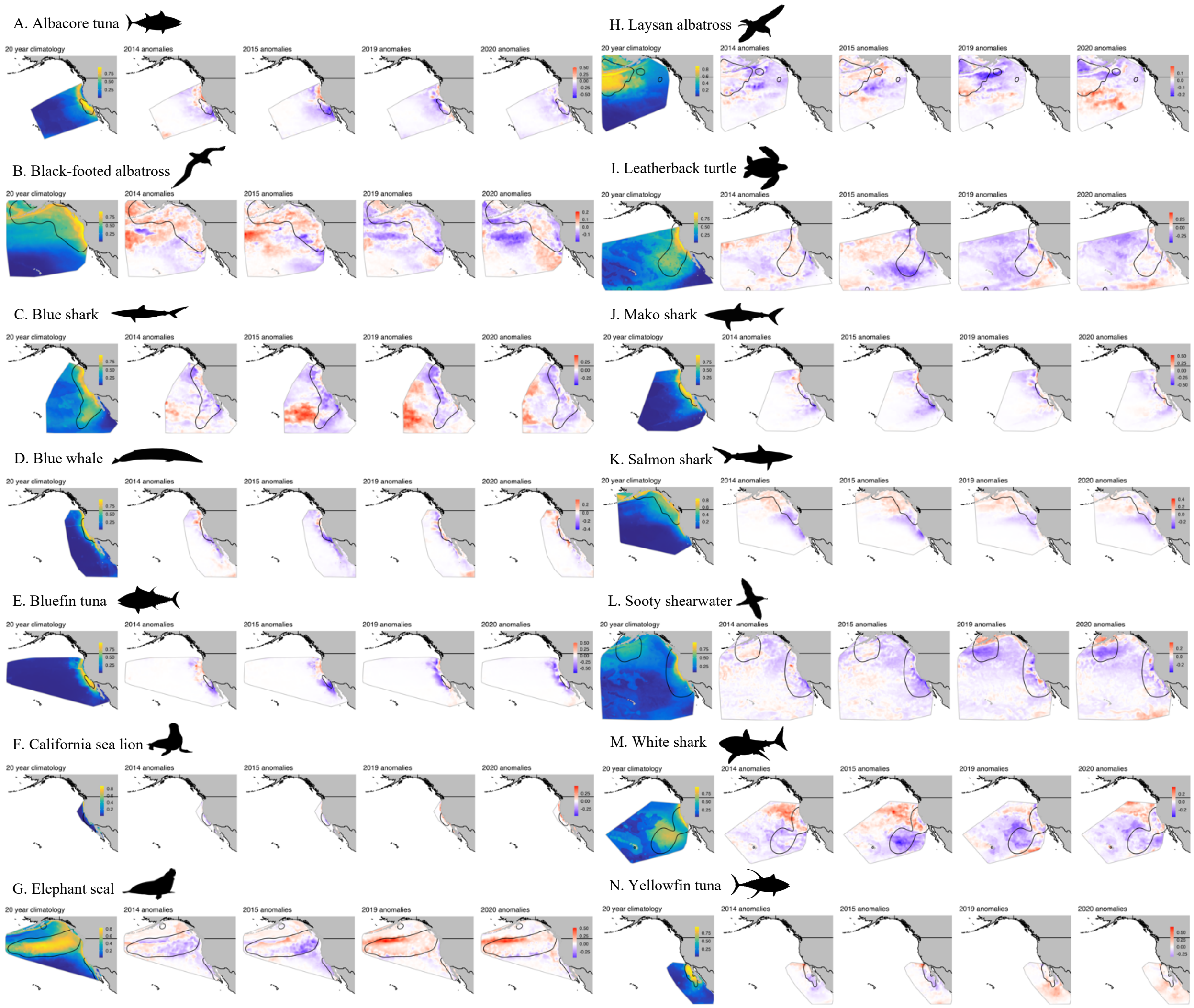

Supplementary Figure 9. Species baseline distributions and distributions during marine heatwaves. Baseline distributions (August-October 2000-2020 average probability of presence), and distribution anomalies for each marine heatwave event (August-October of each year) for each species. Black contour shows 75th percentile kernels for each species based on all telemetry dataset observations in August-October.

1.5.7. Model caveats

While species distribution models have become a standard tool in the spatial ecology toolkit, they are sensitive to choices made during the model fitting process. A central assumption is that statistical relationships effectively capture the underlying mechanisms driving species distributions and habitat use. In addition, we assume that these relationships remain stationary as species encounter novel environmental conditions. The choice of model type will also affect species-environment relationships and spatial predictions, with BRTs typically producing more irregular response curves and predictions than more frequentist models such as Generalized Additive Models[40]. The environmental data selected as model covariates will affect the types of processes that can be approximated, and the spatio-temporal resolution of environmental data will affect the ability of models to capture fine-scale ephemeral features such as filaments and eddies. Background pseudo-absence sampling, as used in this analysis, will capture broadscale habitat use and is an effective way in which to build robust presence-absence models[32]. The ratio of presences to pseudo-absences affects the prevalence of species in model predictions, and here we

used the standard 1:1 approach that is best for BRTs[33]. Finally, the threshold used to define core habitat can be adjusted to prioritize model specificity or sensitivity, and our threshold of 50% provided equal weighting and also allowed for core habitat during MHW years. Our validation data set consisting of records from eight different sampling programs represents the gold standard for validation data[41], however there are some species and MHWs for which we do not have validation data. These decision points exist in every analysis that uses species distribution models (aside from the core habitat threshold), and as such a number of best practice guides have been written to guide model fit[32,33,35,41,42]. At each decision point, we have taken careful steps to follow the best practices for our cross-specific data and model purpose while documenting the decisions to ensure reproducibility.

1.6. Quantifying MHW impacts

1.6.1. Habitat metrics

Three habitat metrics were calculated from the daily binary core habitat rasters: the centroid of core habitat (mean latitude/longitude coordinate pairs), the interquartile range of core habitat (latitude/longitude coordinate pairs for the north-south and east-west interquartile ranges), and the total amount of core habitat (km^2). To quantify species' range extents, the daily north-south interquartile range was multiplied by the daily east-west interquartile range (both measured in decimal degrees). The interquartile range was selected over the absolute range to remove the effect of outliers (e.g. individual pixels of core habitat with extreme distributions), and to ensure that expansion of range was not artificially truncated due to proximity to the convex hull boundary.

The three metrics were averaged across August-October in each MHW year (2014, 2015, 2019, 2020), and deviations from the 2000-2020 mean were calculated for each habitat metric and each species, producing estimates of habitat displacement, changes to the range extent, and changes to the habitat area. Habitat displacement was assessed as a vector (i.e., cardinal direction and distance) joining the long-term mean position of the core habitat centroid to its MHW-specific location. Range extent change reflected the MHW-specific compression or expansion of range extent compared to the mean range, measured by area, while habitat area change assessed the MHW-specific loss or gain of total core habitat, measured by area. In addition to species-specific habitat metrics (Fig. 2, Fig. 3, Supplementary Fig. 10), the mean and standard deviation of each metric was calculated across the regional species groups (Supplementary Table 4).

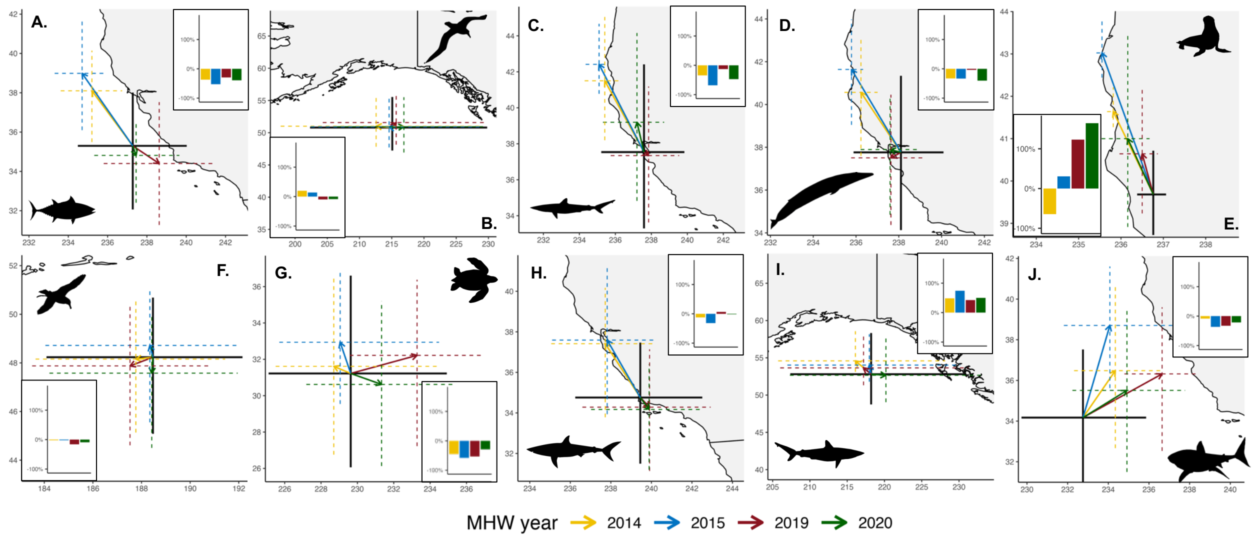

Supplementary Figure 10. Individual species case studies of marine heatwave (MHW) impacts. A. Albacore tuna, B. Black-footed albatross, C. Blue shark D. Blue whale, E. California sea lion, F. Laysan Albatross, G. Leatherback turtle, H. Mako shark, I. Salmon shark, J. White shark. Large maps: arrows indicate predicted habitat displacement from the center of gravity in each MHW (center of colored crosses) relative to the center of gravity during baseline conditions (center of black cross). Crosses indicate predicted longitudinal and latitudinal range extents during each MHW and during baseline conditions (colored and black crosses, respectively); an increase in cross size during a MHW compared to baseline conditions indicates range expansion with the converse representing compression. Inset: percent change habitat area relative to baseline conditions.

Supplementary Table 4. Summary of species impacts. Average values ± one standard deviation of habitat displacement distance and direction, and percent change range and area for each region and marine heatwave event (MHW). Red text indicates negative percent change for range and area. Source data are provided as a Source Data file.

Species Group	MHW	Displacement		Percent change	
		Distance	Direction	Range	Area
Northern (3 species)	2014	182km ± 95	West-NW (291° ± 27)	12% ± 60	8% ± 33
	2015	137km ± 143	NW-North (328° ± 23)	-12% ± 18	15% ± 40
	2019	161km ± 118	West-NW (306° ± 60)	-3% ± 25	21% ± 43
	2020	177km ± 117	East-SE (122° ± 90)	1% ± 17	18% ± 34
Coastal (8 species)	2014	327km ± 81	NW-North (335° ± 10)	-49% ± 28	-26% ± 32
	2015	427km ± 133	NW-North (337° ± 5)	-59% ± 35	-35% ± 40
	2019	148km ± 166	South-SW (195° ± 101)	44% ± 141	2% ± 52
	2020	170km ± 228	West-NW (294° ± 77)	58% ± 174	-9% ± 72
Southern (2 species)	2014	183km ± 135	NW-North (343° ± 50)	-18% ± 5	-27% ± 23
	2015	347km ± 220	North (360° ± 13)	-32% ± 7	-48% ± 13
	2019	392km ± 30	NE-East (65° ± 8)	-38% ± 5	-43% ± 12
	2020	209km ± 34	NE-East (84° ± 30)	-14% ± 19	-25% ± 2

The absolute magnitude of change to species' range extent and habitat area varied greatly across species, due in part to differences in occurrence extent (e.g., seabirds have large ranges relative to California sea lions). In order to make MHW impacts comparable across species, habitat compression and loss were expressed as a percent change from mean conditions.

Due to the convex curvature of the US west coast, centroids for some species were located on land (Supplementary Fig. 10). While a centroid is coarse and does not necessarily represent where species actually are, it still captures the relative relocations of preferred habitat among species.

1.6.2. Habitat metrics sensitivity

We tested the effect of calculating core habitat centroids on binary core habitat (all core habitat pixels assigned a value of 1) versus continuous core habitat (all core habitat pixels maintain values of continuous habitat suitability predictions). This comparison only affects values of habitat displacement (direction and distance) as range extent change and habitat area change do not utilize centroids. We found minimal differences between the two methods of centroid definition, with displacement distance varying on average by 3km, and displacement direction varying on average by 1 degree (Supplementary Fig. 11).

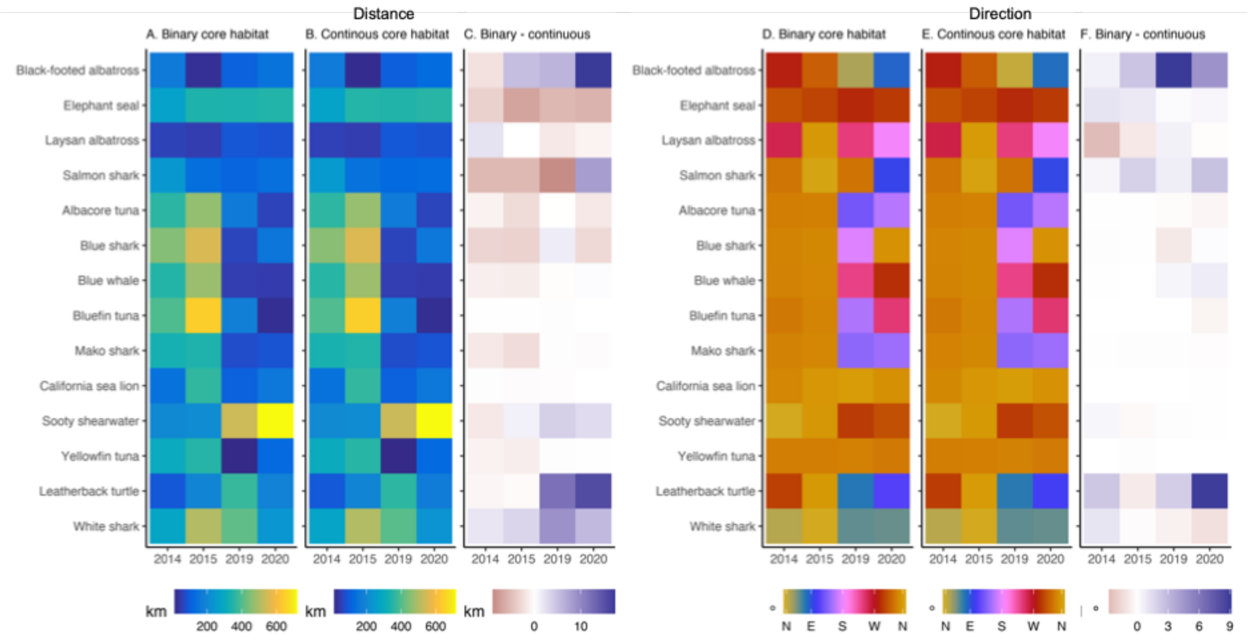

Supplementary Figure 11. Sensitivity of displacement distance (A,B,C) and direction (D,E,F) to core habitat centroids calculated on binary core habitat (all core habitat pixels assigned a value of 1 (A,D)) versus continuous core habitat (all core habitat pixels maintain values of continuous habitat suitability predictions (B,E)). C,F show binary core habitat minus continuous core habitat for habitat displacement distance and direction, respectively. Source data are provided as a Source Data file.

In addition, we tested the sensitivity of habitat metrics to the data used in model fitting. For each species, telemetry and pseudo-absence data were randomly subsampled into 20 different subsets containing 75% of the data and maintaining the 1:1 ratio of presences to pseudo-absences. A BRT model was fit to each subset (n=280 models; 14 species x 20 data subsets), predicted, and summarized to calculate habitat metrics following methods in Sections 1.5.2., 1.5.6., and 1.6.1. Then, habitat metrics were summarized across the 20 models by mean value, standard error, and coefficient of variation (Supplementary Table 5).

Coefficient of variation was included in addition to standard error because values are standardized by the mean, controlling for larger values having larger standard errors. We found low sensitivity of habitat metrics to data used in model fitting. The average standard errors across species and MHW years were: displacement distance: 9.81 (±19.2km 95% confidence interval); displacement direction: 2.75 (±5.39° 95% confidence interval); percent change range: 3.52 (±6.89% 95% confidence interval); and percent change area: 1.68 (±3.29% 95% confidence interval). Displacement distance had the largest standard error, but its confidence interval indicates that error (19.2km) was less than the size of one 25km pixel. The mean coefficient of variations across species and MHW years were also low: displacement distance: 0.24; displacement direction: 0.18; % change range: 1.14; and % change area: 0.31.

Supplementary Table 5. Sensitivity of habitat metrics to data used in model fitting. For each habitat metric, species, and marine heatwave (MHW), the mean, standard error (SE), and coefficient of variation (CV) were calculated across 20 models fit to random 75% subsets of the telemetry and pseudo-absence data. SE and CV columns have been shaded to ease interpretation such that darkest reds indicate the largest value in each column. Source data are provided as a Source Data file.

		Displacement distance			Displacement direction			% change range			% change area		
	**MHW**	*Mean*	*SE*	*CV*	*Mean*	*SE*	*CV*	*Mean*	*SE*	*CV*	*Mean*	*SE*	*CV*
**Albacore tuna**	**2014**	390.5	8.8	0.1	-29.4	0.17	0.03	-54.3	2.32	0.19	-40.4	2.27	0.25
	**2015**	506.5	29.67	0.26	-28	0.4	0.06	-47.9	4.24	0.4	-55.4	2.99	0.24
	**2019**	188.4	4.92	0.12	130.3	0.69	0.02	-3	5.62	8.47	-37.5	2.39	0.29
	**2020**	99.2	9.08	0.41	154.4	3.09	0.09	-48.7	2.91	0.27	-43.3	1.69	0.18
**Black-footed albatross**	**2014**	155.1	7.51	0.22	-80.7	0.85	0.05	8.9	2.24	1.13	14.6	0.85	0.26
	**2015**	45.8	7.47	0.73	-49.9	12	1.08	-8.6	0.62	0.32	8.9	0.74	0.37
	**2019**	103.6	2.68	0.12	24	4.24	0.79	-14.7	0.88	0.27	-9.5	0.74	0.35
	**2020**	130.3	7.14	0.25	73.1	3.84	0.24	-13.2	1.05	0.36	-10.8	0.86	0.36
**Blue shark**	**2014**	422.9	22.62	0.24	-26	0.35	0.06	-65.2	0.74	0.05	-34.9	0.95	0.12
	**2015**	518.7	26.23	0.23	-24.7	0.49	0.09	-83.1	1.92	0.1	-64.8	1.16	0.08
	**2019**	70.9	10.67	0.67	-177.2	11.3	0.29	-20.6	1.65	0.36	-17.4	1.06	0.27
	**2020**	159.3	18.22	0.51	-5	10.87	9.76	-14.1	2.38	0.75	-45.9	1.75	0.17
**Blue whale**	**2014**	364.4	19.28	0.23	-27.3	0.22	0.04	-72.2	1	0.06	-34.1	1.2	0.15
	**2015**	467.2	21.63	0.2	-25.6	0.26	0.04	-79.7	1.75	0.1	-35	2.65	0.33
	**2019**	90.2	6.34	0.31	-150	7.59	0.22	-46.9	2.06	0.19	-12.5	2.21	0.77
	**2020**	95	12.72	0.58	-127.6	14.18	0.48	-58.7	3.69	0.27	-44.7	2.53	0.25
**Bluefin tuna**	**2014**	395.4	2.28	0.03	-33.5	0.08	0.01	-63.3	0.51	0.04	-39.2	0.39	0.04
	**2015**	636.5	10.3	0.07	-26.8	0.22	0.04	-88.9	1.98	0.1	-94.5	0.3	0.01
	**2019**	183.3	3.8	0.09	153.2	0.51	0.01	-56.9	2.18	0.17	-47.6	1.38	0.13
	**2020**	32.9	3.29	0.45	-112.9	10.41	0.41	-42.7	1.73	0.18	-63.3	1.15	0.08
**California sea lion**	**2014**	234.6	10.06	0.19	-19.1	0.37	0.09	-91.9	1.38	0.07	-69.2	1.47	0.09
	**2015**	386.9	5.03	0.06	-14.7	0.27	0.08	-82.9	1.23	0.07	10.7	4.75	1.99
	**2019**	88.7	9.57	0.48	-15.5	1.16	0.33	184.6	24.27	0.59	152.7	9.55	0.28
	**2020**	129	7.76	0.27	-20.9	0.76	0.16	338.8	25.21	0.33	191.4	12.76	0.3
**Elephant seal**	**2014**	270.6	3.38	0.06	-52.3	0.73	0.06	99.2	1.97	0.09	-29	0.37	0.06
	**2015**	338.5	3.91	0.05	-59.6	0.66	0.05	10.2	1.24	0.54	-17.6	0.37	0.09
	**2019**	345.8	4.14	0.05	-76.5	0.36	0.02	29.4	0.78	0.12	69.2	0.96	0.06
	**2020**	349.1	5.04	0.06	-67.1	0.26	0.02	23.5	0.54	0.1	43.9	1.04	0.11
**Laysan albatross**	**2014**	60.7	3.78	0.28	-97	5.19	0.24	-4.5	1.58	1.57	-5.7	1.2	0.94
	**2015**	44.6	2.17	0.22	-24.2	3.02	0.56	-8	0.46	0.26	-6.9	0.73	0.47
	**2019**	104.8	4.02	0.17	-126.8	1.66	0.06	-7.9	1.62	0.91	-18.3	0.51	0.12
	**2020**	92.1	3.01	0.15	-177.1	1.47	0.04	-8.5	0.98	0.51	-11.1	0.72	0.29
**Leatherback turtle**	**2014**	100.5	5.98	0.27	-60.6	3.18	0.23	-9.8	1.57	0.71	-10.1	0.39	0.17
	**2015**	269.3	11.5	0.19	-16.2	3.46	0.96	-31.4	2.75	0.39	-37.3	1.69	0.2
	**2019**	416.4	23.62	0.25	77.6	1.73	0.1	-32.1	3.57	0.5	-33.5	1.51	0.2
	**2020**	241.1	14.22	0.26	100.7	4.09	0.18	-27.3	2.06	0.34	-25.1	0.76	0.14
**Mako shark**	**2014**	339.8	7.66	0.1	-27.5	0.07	0.01	-1.6	6.77	18.88	-15.9	0.95	0.27
	**2015**	358.8	11.57	0.14	-24.8	0.33	0.06	12	6.09	2.26	-35.3	1.34	0.17
	**2019**	69.9	3.79	0.24	140.2	0.76	0.02	9.1	3.52	1.72	6.3	1.45	1.04
	**2020**	83.5	3.48	0.19	148.2	0.6	0.02	-22.1	1.59	0.32	-2.1	0.69	1.48
**Salmon shark**	**2014**	202.9	2.72	0.06	-50.5	0.32	0.03	-31.9	0.61	0.09	7	0.65	0.41
	**2015**	116.4	4.58	0.18	-42.9	1.11	0.12	-20.9	0.58	0.12	18.7	0.79	0.19
	**2019**	29	3.38	0.52	-108.1	9.48	0.39	-5.4	0.73	0.6	13.8	0.63	0.2
	**2020**	83.9	7.19	0.38	119.3	1.54	0.06	0.4	0.83	8.4	12.1	1.06	0.39
**Sooty shearwater**	**2014**	233.8	21.76	0.42	9	7.4	3.66	-39.3	5.14	0.58	-30.5	0.96	0.14
	**2015**	250.1	11.34	0.2	-13.9	2.37	0.76	-53.4	3.67	0.31	-38.3	0.95	0.11
	**2019**	453.5	27.27	0.27	-71.3	1.87	0.12	280.4	23.02	0.37	-25.8	2.78	0.48
	**2020**	717.5	39.11	0.24	-55.8	0.75	0.06	295.8	20.94	0.32	-43.6	3.12	0.32
**White shark**	**2014**	234.5	7.39	0.14	20.2	1.77	0.39	-19.8	1.27	0.29	-38.4	0.7	0.08
	**2015**	446.7	8.95	0.09	15.6	0.85	0.24	-40.4	3.52	0.39	-51.5	1.13	0.1
	**2019**	345.3	17.54	0.23	59.5	1.24	0.09	-44.9	1.13	0.11	-43.1	1.87	0.19
	**2020**	203.8	10.44	0.23	60.2	1.88	0.14	1	1.7	7.41	-22.5	1.23	0.24
**Yellowfin tuna**	**2014**	304.3	3.14	0.05	-30.4	0.08	0.01	-22.3	0.88	0.18	42	1.96	0.21
	**2015**	346.3	3.62	0.05	-28.8	0.18	0.03	-14.1	1.05	0.33	13.8	1.68	0.55
	**2019**	8.2	1.16	0.63	-52.2	11.07	0.95	-14.4	0.47	0.14	18	1.13	0.28
	**2020**	123.7	1.17	0.04	-33.1	0.22	0.03	-28.6	0.67	0.1	13.9	0.83	0.27

1.6.3. Variability in habitat metrics within vs among MHWs

We explored the extent to which variability in habitat metrics was associated with species vs MHWs using linear mixed effect models. For each habitat metric (distance, percent change range, percent change area), we built two models with one fixed effect and one random effect, e.g. for percent change area:

Mod1: lmer(percent change area ~ MHW + (1|species), data = dat)
Mod2: lmer(percent change area~ species + (1| MHW), data = dat)

In Mod1, MHW is fixed and species is random, and in Mod2, MHW is random and species is fixed. Next, we compared the marginal R^2 values for each model, i.e. the R^2 considering only the fixed effect and ignoring the random effect. This comparison allowed us to determine if MHW explains more variance while controlling for species (Mod1), or if species explains more variance while controlling for MHW (Mod2) (Supplementary Table 6).

Supplementary Table 6. Linear mixed models for percent change range, percent change area, and distance habitat metrics. Each model contained one random effect (either species or marine heatwave (MHW)) and one fixed effect. Marginal R^2 values indicate the variance explained by the fixed effect while controlling for the random effect.

Metric	Random effect	Fixed effect	Marginal R^2	Tests
Percent change range	Species	MHW	0.1	The variance explained by MHW
Percent change range	MHW	Species	0.31	The variance explained by species
Percent change area	Species	MHW	0.04	The variance explained by MHW
Percent change area	MHW	Species	0.55	The variance explained by species
Distance	Species	MHW	0.13	The variance explained by MHW
Distance	MHW	Species	0.25	The variance explained by species

Results indicate that species explains more of the variance than MHW for percent change range, percent change area, and distance habitat metrics.

1.7. Cross-jurisdictional impacts

We quantified how species core habitats redistributed across national jurisdictions during MHW events. Version 11 of Exclusive Economic Zone (EEZ) boundaries was used to define national jurisdictions (marineregions.org)[43] (Supplementary Fig. 4). The amount of each species core habitat within the EEZs of Canada, the US, and Mexico, and within the high seas was calculated for each day in August-October of 2000-2020. For each species, average core habitat area in each jurisdiction was calculated for August-October in each MHW year, and the August-October 2000-2020 baseline was subtracted to obtain the anomaly. Anomalies were expressed as a percent change relative to baseline conditions. Habitat redistribution was summarized as both a percent change for each jurisdiction/MHW (Fig. 4A), and as the largest percent loss and gain for each species (Fig. 4B).

Similar to the habitat metrics, we tested the sensitivity of species core habitat redistribution across jurisdictions to the data used in model fitting by fitting and predicting 20 replicate BRT models (Section 1.6.2.). Habitat redistributions were calculated as above, and summarized across the 20 models by mean anomaly, standard error, and coefficient of variation (Supplementary Table 7). The average standard errors and coefficients of variations across species, MHW years, and jurisdictions were low: 0.0031 (±.0061% 95% confidence interval) and 1.47 for standard error and coefficient of variation, respectively.

Supplementary Table 7. Sensitivity of species core habitat redistribution across jurisdictions to data used in model fitting. For each jurisdiction, species, and marine heatwave (MHW), the mean anomaly, standard error (SE), and coefficient of variation (CV) were calculated across 20 models fit to random 75% subsets of the telemetry and pseudo-absence data. SE and CV columns have been shaded to ease interpretation such that darkest reds indicate the largest value in each column. Source data are provided as a Source Data file.

		Canadian EEZ			High seas			US EEZ			Mexican EEZ		
	**MHW**	*Mean*	*SE*	*CV*	*Mean*	*SE*	*CV*	*Mean*	*SE*	*CV*	*Mean*	*SE*	*CV*
**Albacore tuna**	2014	0.00%	NA	NA	0.31%	0.0008	1.1	17.06%	0.0046	0.12	-17.37%	0.0048	0.12
	2015	0.00%	NA	NA	-2.57%	0.0019	0.32	20.24%	0.0062	0.14	-17.67%	0.0050	0.13
	2019	0.00%	NA	NA	-2.28%	0.0021	0.41	-6.61%	0.0077	0.52	8.89%	0.0082	0.41
	2020	0.00%	NA	NA	-3.01%	0.0027	0.4	7.60%	0.0139	0.82	-4.59%	0.0125	1.22
**Black-footed albatross**	2014	-1.56%	0.0010	0.29	1.58%	0.0027	0.75	-0.02%	0.0030	74.39	0.00%	NA	NA
	2015	-0.39%	0.0011	1.26	4.38%	0.0031	0.31	-3.99%	0.0031	0.35	0.00%	NA	NA
	2019	1.23%	0.0013	0.47	0.25%	0.0061	11.12	-1.48%	0.0065	1.98	0.00%	NA	NA
	2020	1.50%	0.0021	0.63	2.07%	0.0052	1.14	-3.57%	0.0061	0.77	0.00%	NA	NA
**Blue shark**	2014	0.75%	0.0025	1.48	-0.48%	0.0017	1.55	9.89%	0.0027	0.12	-10.15%	0.0024	0.11
	2015	-0.11%	0.0038	15.42	-0.75%	0.0021	1.25	11.45%	0.0041	0.16	-10.59%	0.0027	0.11
	2019	-2.30%	0.0028	0.55	-0.77%	0.0011	0.64	6.39%	0.0045	0.31	-3.33%	0.0071	0.95
	2020	-1.60%	0.0032	0.9	-0.81%	0.0013	0.73	6.77%	0.0039	0.26	-4.35%	0.0065	0.67
**Blue whale**	2014	0.74%	0.0013	0.76	-2.81%	0.0100	1.59	17.18%	0.0216	0.56	-15.12%	0.0122	0.36
	2015	1.18%	0.0010	0.37	-1.99%	0.0011	0.25	15.32%	0.0082	0.24	-14.50%	0.0077	0.24
	2019	-1.07%	0.0016	0.67	-2.42%	0.0102	1.89	15.87%	0.0215	0.6	-12.38%	0.0122	0.44
	2020	-0.14%	0.0045	14.03	-2.06%	0.0103	2.24	12.86%	0.0230	0.8	-10.66%	0.0127	0.53
**Bluefin tuna**	2014	0.00%	NA	NA	-0.19%	0.0002	0.43	10.90%	0.0044	0.18	-10.71%	0.0043	0.18
	2015	0.00%	NA	NA	-0.19%	0.0002	0.43	10.78%	0.0036	0.15	-10.59%	0.0034	0.15
	2019	0.00%	NA	NA	-0.16%	0.0001	0.24	0.89%	0.0060	3.03	-0.73%	0.0060	3.69
	2020	0.00%	NA	NA	-0.19%	0.0002	0.39	4.70%	0.0041	0.39	-4.51%	0.0041	0.41
**California sea lion**	2014	0.00%	NA	NA	-0.82%	0.0012	0.63	0.92%	0.0014	0.67	-0.09%	0.0006	2.77
	2015	0.00%	NA	NA	-0.81%	0.0011	0.62	0.90%	0.0013	0.65	-0.09%	0.0006	2.77
	2019	0.00%	NA	NA	-0.15%	0.0009	2.82	0.24%	0.0011	2.11	-0.09%	0.0006	2.77
	2020	0.00%	NA	NA	-0.67%	0.0010	0.69	0.75%	0.0012	0.73	-0.08%	0.0006	3
**Elephant seal**	2014	2.34%	0.0006	0.12	-9.23%	0.0015	0.07	7.22%	0.0015	0.09	-0.33%	0.0004	0.49
	2015	2.10%	0.0014	0.29	-2.49%	0.0025	0.45	1.00%	0.0014	0.63	-0.62%	0.0003	0.24
	2019	0.70%	0.0007	0.43	2.96%	0.0013	0.19	-2.60%	0.0007	0.11	-1.07%	0.0002	0.07
	2020	4.38%	0.0010	0.1	-0.44%	0.0013	1.34	-2.99%	0.0007	0.11	-0.94%	0.0002	0.08
**Laysan albatross**	2014	-0.01%	0.0000	0.6	-0.61%	0.0101	7.41	0.62%	0.0101	7.31	0.00%	NA	NA
	2015	-0.01%	0.0000	0.64	-0.30%	0.0052	7.63	0.31%	0.0052	7.36	0.00%	NA	NA
	2019	-0.01%	0.0000	0.59	5.63%	0.0086	0.69	-5.62%	0.0087	0.69	0.00%	NA	NA
	2020	-0.01%	0.0000	0.65	10.28%	0.0075	0.33	-10.27%	0.0075	0.33	0.00%	NA	NA
**Leatherback turtle**	2014	0.00%	NA	NA	3.73%	0.0052	0.62	-1.02%	0.0031	1.36	-2.71%	0.0022	0.36
	2015	0.00%	NA	NA	-2.43%	0.0104	1.92	5.82%	0.0122	0.94	-3.39%	0.0020	0.27
	2019	0.00%	NA	NA	-12.01%	0.0043	0.16	7.96%	0.0044	0.25	4.05%	0.0017	0.18
	2020	0.00%	NA	NA	-8.00%	0.0083	0.46	6.58%	0.0073	0.5	1.42%	0.0014	0.43
**Mako shark**	2014	0.00%	NA	NA	0.37%	0.0002	0.29	13.99%	0.0043	0.14	-14.35%	0.0045	0.14
	2015	0.00%	NA	NA	0.79%	0.0006	0.34	14.48%	0.0062	0.19	-15.27%	0.0065	0.19
	2019	0.00%	NA	NA	-0.04%	0.0002	2.91	-2.66%	0.0015	0.25	2.70%	0.0016	0.26
	2020	0.00%	NA	NA	0.13%	0.0003	1.06	-2.19%	0.0019	0.4	2.06%	0.0018	0.4
**Salmon shark**	2014	-0.56%	0.0006	0.51	-0.18%	0.0003	0.76	0.75%	0.0008	0.45	-0.01%	0.0000	0.72
	2015	4.42%	0.0018	0.18	-0.21%	0.0003	0.72	-4.20%	0.0018	0.19	-0.01%	0.0000	0.72
	2019	1.21%	0.0015	0.54	0.04%	0.0002	2.09	-1.24%	0.0015	0.53	-0.01%	0.0000	0.72
	2020	3.16%	0.0022	0.31	0.32%	0.0003	0.39	-3.46%	0.0022	0.28	-0.01%	0.0000	0.72
**Sooty shearwater**	2014	6.00%	0.0023	0.17	-0.29%	0.0016	2.45	-0.94%	0.0033	1.56	-4.77%	0.0023	0.21
	2015	3.76%	0.0029	0.35	-0.31%	0.0014	2.05	1.69%	0.0034	0.9	-5.15%	0.0019	0.16
	2019	-2.73%	0.0034	0.55	0.14%	0.0024	7.85	6.62%	0.0046	0.31	-4.02%	0.0017	0.19
	2020	1.22%	0.0061	2.25	0.71%	0.0031	1.92	1.17%	0.0095	3.63	-3.10%	0.0017	0.25
**White shark**	2014	0.00%	NA	NA	-15.50%	0.0039	0.11	15.30%	0.0038	0.11	0.19%	0.0004	0.83
	2015	0.00%	NA	NA	-29.67%	0.0092	0.14	29.69%	0.0092	0.14	-0.02%	0.0000	0.78
	2019	0.00%	NA	NA	-33.15%	0.0118	0.16	33.17%	0.0118	0.16	-0.02%	0.0000	1.07
	2020	0.00%	NA	NA	-17.71%	0.0053	0.13	17.55%	0.0053	0.13	0.16%	0.0001	0.41
**Yellowfin tuna**	2014	0.00%	NA	NA	0.29%	0.0003	0.45	22.44%	0.0035	0.07	-22.73%	0.0035	0.07
	2015	0.00%	NA	NA	0.21%	0.0002	0.53	30.31%	0.0033	0.05	-30.52%	0.0033	0.05
	2019	0.00%	NA	NA	0.03%	0.0002	2.95	-10.81%	0.0020	0.08	10.78%	0.0021	0.09
	2020	0.00%	NA	NA	-0.12%	0.0001	0.2	-2.81%	0.0022	0.36	2.93%	0.0022	0.34

Supplementary References

1. Block, B. A. *et al.* Tracking apex marine predator movements in a dynamic ocean. *Nature* **475**, 86–90 (2011).
2. Hazen, E. L. *et al.* Predicted habitat shifts of Pacific top predators in a changing climate. *Nat. Clim. Change* **3**, 234–238 (2013).
3. Nasby-Lucas, N., Dewar, H., Lam, C. H., Goldman, K. J. & Domeier, M. L. White Shark Offshore Habitat: A Behavioral and Environmental Characterization of the Eastern Pacific Shared Offshore Foraging Area. *PLOS ONE* **4**, e8163 (2009).
4. Scannell, H. A., Johnson, G. C., Thompson, L., Lyman, J. M. & Riser, S. C. Subsurface Evolution and Persistence of Marine Heatwaves in the Northeast Pacific. *Geophys. Res. Lett.* **47**, e2020GL090548 (2020).
5. Jacox, M. G. *et al.* Impacts of the 2015–2016 El Niño on the California Current System: Early assessment and comparison to past events. *Geophys. Res. Lett.* **43**, 7072–7080 (2016).
6. Rudnick, D. L., Zaba, K. D., Todd, R. E. & Davis, R. E. A climatology of the California Current System from a network of underwater gliders. *Prog. Oceanogr.* **154**, 64–106 (2017).
7. Amaya, D. J., Miller, A. J., Xie, S.-P. & Kosaka, Y. Physical drivers of the summer 2019 North Pacific marine heatwave. *Nat. Commun.* **11**, 1903 (2020).
8. Weber, E. D. *et al.* State of the California Current 2019–2020: Back to the Future With Marine Heatwaves? *Front. Mar. Sci.* **8**, 1081 (2021).
9. Hobday, A. J. *et al.* A hierarchical approach to defining marine heatwaves. *Prog. Oceanogr.* **141**, 227–238 (2016).
10. Winship, A. J. *et al.* State-space framework for estimating measurement error from double-tagging telemetry experiments. *Methods Ecol. Evol.* **3**, 291–302 (2012).
11. Gutowsky, S. E. *et al.* Daily activity budgets reveal a quasi-flightless stage during non-breeding in Hawaiian albatrosses. *Mov. Ecol.* **2**, 23 (2014).
12. Jordan, F. D. *et al.* Divergent post-breeding spatial habitat use of Laysan and black-footed albatross. *Front. Ecol. Evol.* **10**, (2022).
13. Shaffer, S. A. *et al.* Comparison of light- and SST-based geolocation with satellite telemetry in free-ranging albatrosses. *Mar. Biol.* **147**, 833–843 (2005).
14. Conners, M. G., Hazen, E. L., Costa, D. P. & Shaffer, S. A. Shadowed by scale: subtle behavioral niche partitioning in two sympatric, tropical breeding albatross species. *Mov. Ecol.* **3**, 28 (2015).
15. Weimerskirch, H., Pinaud, D., Pawlowski, F. & Bost, C. Does Prey Capture Induce Area-Restricted Search? A Fine-Scale Study Using GPS in a Marine Predator, the Wandering Albatross. *Am. Nat.* **170**, 734–743 (2007).
16. Bailey, H. *et al.* Behavioural estimation of blue whale movements in the Northeast Pacific from state-space model analysis of satellite tracks. *Endanger. Species Res.* **10**, 93–106 (2009).
17. Boustany, A. M., Matteson, R., Castleton, M., Farwell, C. & Block, B. A. Movements of pacific bluefin tuna (Thunnus orientalis) in the Eastern North Pacific revealed with archival tags. *Prog. Oceanogr.* **86**, 94–104 (2010).
18. Carroll, G. *et al.* Flexible use of a dynamic energy landscape buffers a marine predator against extreme climate variability. *Proc. R. Soc. B Biol. Sci.* **288**, 20210671 (2021).
19. Muhling, B. A. *et al.* Risk and Reward in Foraging Migrations of North Pacific Albacore Determined From Estimates of Energy Intake and Movement Costs. *Front. Mar. Sci.* **9**, (2022).
20. Maxwell, S. M. *et al.* Seasonal spatial segregation in blue sharks (Prionace glauca) by sex and size class in the Northeast Pacific Ocean. *Divers. Distrib.* **25**, 1304–1317 (2019).
21. Briscoe, D. K. *et al.* Characterizing habitat suitability for a central-place forager in a dynamic marine environment. *Ecol. Evol.* **8**, 2788–2801 (2018).
22. Żydelis, R. *et al.* Dynamic habitat models: using telemetry data to project fisheries bycatch. *Proc. R. Soc. B Biol. Sci.* **278**, 3191–3200 (2011).
23. Nieto, K., Xu, Y., Teo, S. L. H., McClatchie, S. & Holmes, J. How important are coastal fronts to albacore tuna (Thunnus alalunga) habitat in the Northeast Pacific Ocean? *Prog. Oceanogr.* **150**, 62–

71 (2017).
24. Childers, J., Snyder, S. & Kohin, S. Migration and behavior of juvenile North Pacific albacore (Thunnus alalunga). *Fish. Oceanogr.* **20**, 157–173 (2011).
25. eBird. eBird: An online database of bird distribution and abundance. Cornell Lab of Ornithology, Ithaca, New York. http://www.ebird.org (2021).
26. Drew, G. S. & Piatt, J. F. North Pacific Pelagic Seabird Database (NPPSD). (2015) doi:10.5066/F7WQ01T3.
27. Palacios, D. M. *et al.* Ecological correlates of blue whale movement behavior and its predictability in the California Current Ecosystem during the summer-fall feeding season. *Mov. Ecol.* **7**, 26 (2019).
28. NOAA Alaska Fisheries Science Center. North Pacific (NORPAC) Groundfish and Halibut Observer Data Dictionary December 2007 –Present. https://www.fisheries.noaa.gov/inport/item/7290.
29. Le Boeuf, B. J. *et al.* Foraging Ecology of Northern Elephant Seals. *Ecol. Monogr.* **70**, 353–382 (2000).
30. Robinson, P. W. *et al.* Foraging Behavior and Success of a Mesopelagic Predator in the Northeast Pacific Ocean: Insights from a Data-Rich Species, the Northern Elephant Seal. *PLOS ONE* **7**, e36728 (2012).
31. Strimas-Mackey, M. *et al. Best Practices for Using eBird Data v1.0*. (Zenodo, 2020). doi:10.5281/zenodo.3620739.
32. Hazen, E. L. *et al.* Where did they not go? Considerations for generating pseudo-absences for telemetry-based habitat models. *Mov. Ecol.* **9**, 5 (2021).
33. Barbet-Massin, M., Jiguet, F., Albert, C. H. & Thuiller, W. Selecting pseudo-absences for species distribution models: how, where and how many? *Methods Ecol. Evol.* **3**, 327–338 (2012).
34. White, T. D. *et al.* Predicted hotspots of overlap between highly migratory fishes and industrial fishing fleets in the northeast Pacific. *Sci. Adv.* **5**, eaau3761 (2019).
35. Elith, J., Leathwick, J. R. & Hastie, T. A working guide to boosted regression trees. *J. Anim. Ecol.* **77**, 802–813 (2008).
36. Cimino, M. A., Anderson, M., Schramek, T., Merrifield, S. & Terrill, E. J. Towards a Fishing Pressure Prediction System for a Western Pacific EEZ. *Sci. Rep.* **9**, 461 (2019).
37. Crespo, G. O. *et al.* The environmental niche of the global high seas pelagic longline fleet. *Sci. Adv.* **4**, eaat3681.
38. Becker, E. A. *et al.* Predicting cetacean abundance and distribution in a changing climate. *Divers. Distrib.* **25**, 626–643 (2019).
39. Robinson, L. M., Hobday, A. J., Possingham, H. P. & Richardson, A. J. Trailing edges projected to move faster than leading edges for large pelagic fish habitats under climate change. *Deep Sea Res. Part II Top. Stud. Oceanogr.* **113**, 225–234 (2015).
40. Brodie, S. J. *et al.* Trade-offs in covariate selection for species distribution models: a methodological comparison. *Ecography* **43**, 11–24 (2020).
41. Araújo, M. B. *et al.* Standards for distribution models in biodiversity assessments. *Sci. Adv.* **5**, eaat4858 (2019).
42. Elith, J. & Leathwick, J. R. Species Distribution Models: Ecological Explanation and Prediction Across Space and Time. *Annu. Rev. Ecol. Evol. Syst.* **40**, 677–697 (2009).
43. Flanders Marine Institute. Union of the ESRI Country shapefile and the Exclusive Economic Zones (version 3). Available online at https://www.marineregions.org/. https://doi.org/10.14284/403. Consulted on 2023-02-01. (2020).
